# The effect of whole body position on lumbar cerebrospinal fluid opening pressure

**DOI:** 10.1186/1743-8454-5-11

**Published:** 2008-07-02

**Authors:** Pasiri Sithinamsuwan, Nakorn Sithinamsuwan, Sirakarn Tejavanija, Chesda Udommongkol, Samart Nidhinandana

**Affiliations:** 1 Division of Neurology, Phramongkutklao Hospital and College of Medicine, Bangkok, 10400, Thailand; 2 Department of Medicine, Phramongkutklao Hospital and College of Medicine, Bangkok, 10400, Thailand

## Abstract

We compared cerebrospinal fluid (CSF) opening pressure measurements in the lumbar subarachnoid space between the flexed position (F-OP) and relaxed position (R-OP) in recumbent patients. We devised an equation for using F-OP to determine the existence of raised intracranial pressure (ICP). Patients (n = 83) underwent lumbar puncture while in the flexed lateral decubitus position and then were moved to the relaxed position. F-OP and R-OP were measured with a water manometer. R-OP > 180 mmH_2_O plus relevant clinical signs were taken as indicators of raised intracranial pressure. Mean pressures for F-OP and R-OP were 178.54 and 160.52 mmH_2_O respectively, *p *<0.001. When F-OP > 180, raised ICP could be significantly over diagnosed. The authors recommend an equation [R-OP_(calculated, mmH2O) _= 0.885 × F-OP_(measured, mmH2O)_] or using 200 mmH_2_O as the threshold for increased ICP with flexed posture.

## Findings

The existing literature recommends that physicians measure CSF opening pressure with the patient in the recumbent position. A few citations recommend using the relaxed position (neck not flexed, leg extended and without valsalva manoeuvres) because the flexed position is believed to increase lumbar CSF pressure [1-3]. There are very few existing studies available on recommended positions for CSF measurement [4,5]. We have noticed in our clinical practice that many physicians usually measure F-OP rather than R-OP because of the convenience. Our objectives were to determine the effect of the body position on CSF pressure, by comparing F-OP with R-OP in the same patients and to ascertain if there are any differences between the two values. In addition we also investigated the cut off value for raised intracranial pressure, and derived a valid equation for the relation between F-OP and R-OP.

Eighty three consecutive patients were enrolled prospectively and underwent lumbar puncture in the lateral decubitus position between June 1^st^, 2004 and January 31^st^, 2005 in the Phramongkutklao Hospital, Bangkok. The inclusion criteria were: age >14 years, an indication for lumbar puncture for diagnostic or therapeutic purposes and good cooperation from the patient. Exclusion criteria were: pregnancy, contraindication for lumbar puncture, increased intra-thoracic or intra-abdominal pressure (marked ascites or pleural effusion, on ventilator, hyperventilation, cough, sneezing, uncontrolled movement and severe anxiety or agitation), and a marked fall in CSF pressure during measurement. Demographic characteristics were recorded. The mean value of F-OP was measured by a cylindrical glass water manometer after the pressure had stabilized with minimal fluctuation for at least 2 minutes. Patients were supported while they slowly relaxed their posture as much as possible, and R-OP was measured by the same technique. A measurement of R-OP over 180 mmH_2_O, together with clinical signs (several bilateral headaches and/or papilloedema) was used as an indication for increased intracranial pressure. The Medical Department Ethical Committee approved this study in 2004 and patients or responsible relatives all gave written informed consent.

**Figure 1:**
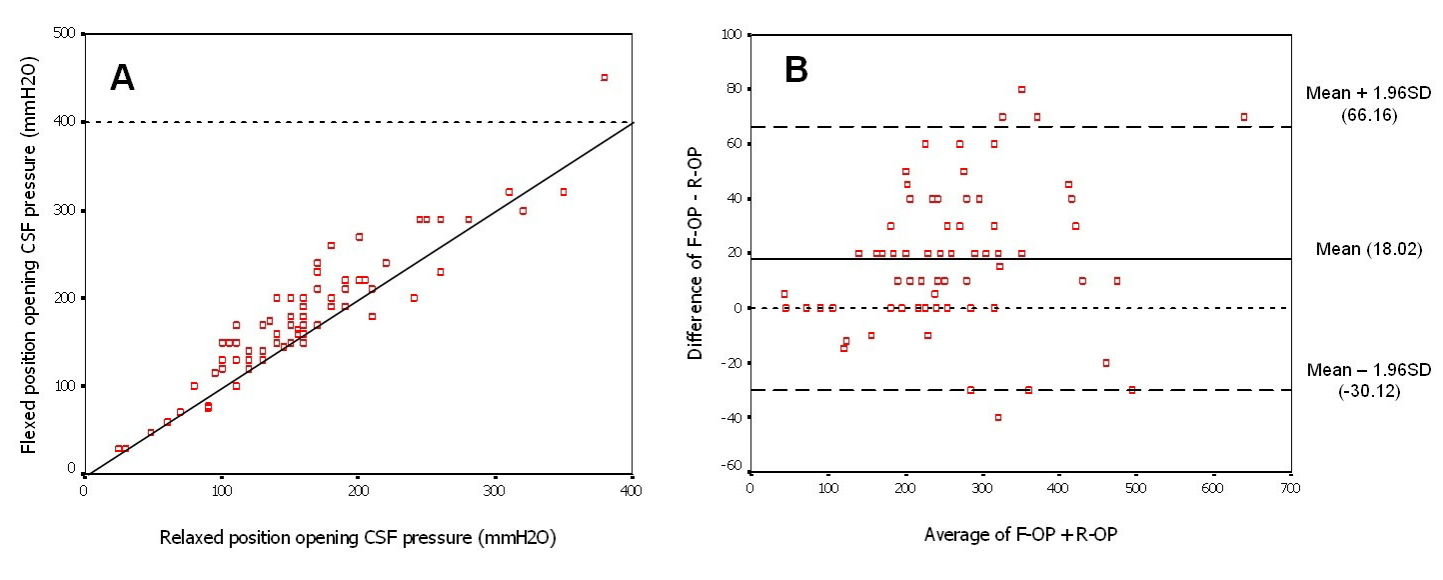
**Correlation and agreement between flexed and relaxed opening CSF pressure measurement in patients in the lateral recumbent position: 1A.** Scatter plot showing the positive correlation between the opening pressure in the flexed position and the opening pressure in the relaxed position; however, the pressure values in flexed position tend to exceed those in the relaxed position as shown in the area between straight line (agreement line) and dot line (showed the F-OP value of 400 mmH_2_O). 1B. Bland-Altman plot showing the relation between the difference between F-OP and R-OP and the mean of F-OP and R-OP. There is a significant disagreement between the pressure values in different positions. The average of the difference (bias) is 18.02 (straight line) compared to the agreement line at zero (dotted) line. In addition, the 95% confidence of the bias is shown as the two dashed lines, which is between -30.12 and 66.16.

Of the 83 patients, 45 were males (54.2%) and 38 females (45.8%) with a mean age of 48.7 years, and range 19–87 years. The means (range) of F-OP and R-OP were 178.54 (30–450) and 160.52 (25–380) mmH_2_O, respectively. By paired sample t-test, the mean difference and standard error between F-OP and R-OP was 18.02 +/- 2.7 mmH_2_O [95%CI: 12.66–23.39], *p *<0.001. Correlation and agreement between F-OP and R-OP measurements are shown in Figure 1A and 1B respectively. The correlation coefficient was 0.94, *p *< 0.001. We used "R-OP > 180 mmH_2_O plus clinical signs" as the "gold standard" for the diagnosis of raised intracranial pressure. The number of patients with clinical signs of increased intracranial pressure who were diagnosed by F-OP > 180 mmH_2_O and "gold standard' is shown in Table 1. There was a significant difference in the number of patients who diagnosed with raised intracranial pressure between using F-OP > 180 mmH_2_O and "gold standard" (*p *< 0.0001). Sensitivity and specificity of F-OP > 180 mmH_2_O for increased intracranial pressure were 95.7% and 83.3%, respectively. Increased intracranial pressure was significantly over-diagnosed in 10/60 cases (16.7%) when F-OP > 180 mmH_2_O was used (Table 1). The receiver operating characteristic curve of F-OP cut off for determining increased intracranial pressure was 200 mmH_2_O and had an area under the curve of 0.961 (95% CI of 0.925–0.997, *p *< 0.01). By stepwise linear regression analysis, the equation between F-OP and R-OP was found to be: R-OP = 0.885 × F-OP, *p *< 0.001.

**Table 1:** Numbers of patients with or without clinical signs of increased intracranial pressure when flexed opening pressure was greater or less than 180 mmH_2_O

	Signs of increased ICP (n = 23)	No signs of increased ICP (n = 60)	Total (n = 83)	*p*-value
F-OP > 180 mmH_2_O	22	10	32	< 0.0001
F-OP ≤ 180 mmH_2_O	1	50	51	

When this equation was used to calculate a new R-OP from F-OP, there was a significant improvement for specificity in determining raised intracranial pressure from 83.3% to 93.3%, *p *< 0.001. This calculation also decreased the difference in mean opening pressures between positions from 18.02 to -2.48 mmH_2_O [95% CI for mean value (-7.46)-(2.49), ns, paired sample t-test].

Eighteen per cent of patients had the same value for F-OP and R-OP. Furthermore; 14.7% had an F-OP less than R-OP. Interestingly, all patients aged >60 years and BMI ≤ 20 showed the same value of F-OP and R-OP. Among subjects who had the same value of F-OP and R-OP, we found people of older age group and those with low BMI. Our hypothesis is that the older people would be more relaxed and those with a lower BMI would be less likely to have increased intra-abdominal pressure.

It is concluded that F-OP should not be routinely used in clinical practice as it can falsely diagnose raised intracranial pressure. If physicians routinely prefer to use F-OP, we suggest the cut off at 200 mmH_2_O for diagnosis of raised intracranial pressure and recommend an equation that can estimate R-OP from F-OP [R-OP_(calculated, mmH2O) _= 0.885 × F-OP_(measured, mmH2O)_].

## Competing interests

The authors declare that they have no competing interests.

## Authors' contributions

PS conceived of the study, participated in the study design, coordinated, performed the statistic analysis, and helped to draft the manuscript. NS participated in the study design, coordinated, assisted in all experiments, and helped to draft the manuscript. ST participated in the study design, assisted in all experiments, and involved in drafting the manuscript. CU participated in the study design, and helped to draft the manuscript. SN conceived of the study, participated in the study design, assisted for statistic analysis, and involved in the preparation of the manuscript. All authors have read and approved the final version.
